# Controllable Swarming and Assembly of Micro/Nanomachines

**DOI:** 10.3390/mi9010010

**Published:** 2017-12-29

**Authors:** Conghui Liu, Tailin Xu, Li-Ping Xu, Xueji Zhang

**Affiliations:** Research Center for Bioengineering and Sensing Technology, School of Chemistry and Biological Engineering, University of Science and Technology Beijing, Beijing 100083, China; conghuiliu@yeah.net (C.L.); xuliping@ustb.edu.cn (L.-P.X.)

**Keywords:** collective behavior, micro/nanomotors, swarming behavior

## Abstract

Motion is a common phenomenon in biological processes. Major advances have been made in designing various self-propelled micromachines that harvest different types of energies into mechanical movement to achieve biomedicine and biological applications. Inspired by fascinating self-organization motion of natural creatures, the swarming or assembly of synthetic micro/nanomachines (often referred to micro/nanoswimmers, micro/nanorobots, micro/nanomachines, or micro/nanomotors), are able to mimic these amazing natural systems to help humanity accomplishing complex biological tasks. This review described the fuel induced methods (enzyme, hydrogen peroxide, hydrazine, et al.) and fuel-free induced approaches (electric, ultrasound, light, and magnetic) that led to control the assembly and swarming of synthetic micro/nanomachines. Such behavior is of fundamental importance in improving our understanding of self-assembly processes that are occurring on molecular to macroscopic length scales.

## 1. Introduction

Collective behavior, involving in cooperative arrangements of a variety of individuals, is a common phenomenon that almost exists on all scales ranging from molecules to galaxies [1,2,3]. In nature, most of us must have been fascinated by macroscopic flocks of animals (fishes, ants, birds, grasshopper, human, etc.) or microscopic swarming of self-propelled bacteria or cells in the biological world. For example, in the macroscopic world, schools of fish can move in a rather orderly fashion or change direction amazingly abruptly; collective of ants can work together to accomplish complex carrying tasks. In the microscopic world, swarming of bacteria is great and helpful to access new sources of nutrients, to increase the size of the community, and to colonize niches [4]. The collective behavior of swarms often reflects a change of global conditions. The motion of individuals may be affected by “signals” that are transmitted by other individuals, causing spatial–temporal patterns. The study of simple collective behavior of micro/nanoscale particles can help us to better understand more complex collective systems.

Micro/nanomachines are microscale or nanoscale devices (range from dozens of nanometers to hundreds of micrometers) that are capable of converting energy into movements and forces. These synthetic micro/nanomachines can be propelled by various external chemical stimuli (hydrogen peroxide, hydrazine, etc.) and fuel-free stimuli (electric, light, ultrasound, magnetic) on an ultra-small scale [5,6,7,8,9,10,11,12,13,14,15,16,17,18,19,20,21,22,23,24], which are promising to perform complicated biomedical tasks, such as targeted drug delivery, cell manipulation and isolation, noninvasive surgery, bioimaging or biosensing, environmental monitoring and remediation, and in vivo applications [25,26,27,28,29,30]. In recent years, the swarming or collective behaviors have inspired researchers to design synthetic nanomachines that can cooperate with each other to achieve much complex biological or environmental tasks [31,32,33,34]. The ability to regulate the collective behavior of synthetic nanomotors offers considerable promise for creating groups of machines, which can perform cooperative tasks that would be impossible using a single nanomotor [3,5,32].

In the present review, following an approach that is meant to be as much tutorial and non-technical as possible, we present recent progress in controlling the swarming and the assembly of micro/nanomachines developed in our laboratory and by other research groups. We start from the basic natural collective behavior in the aspects of theories and experiments to identify the different features, leading to self-propulsion of microscopic particles in fluid environments, and then summarize the fuel-induced (enzyme, hydrogen peroxide, hydrazine, etc.) or fuel-free induced (electrical, ultrasound, magnetic, light) collective effects and their related biological applications; finally, we outlook the future directions of this field.

## 2. Collective Behavior in Nature

**Figure 1:**
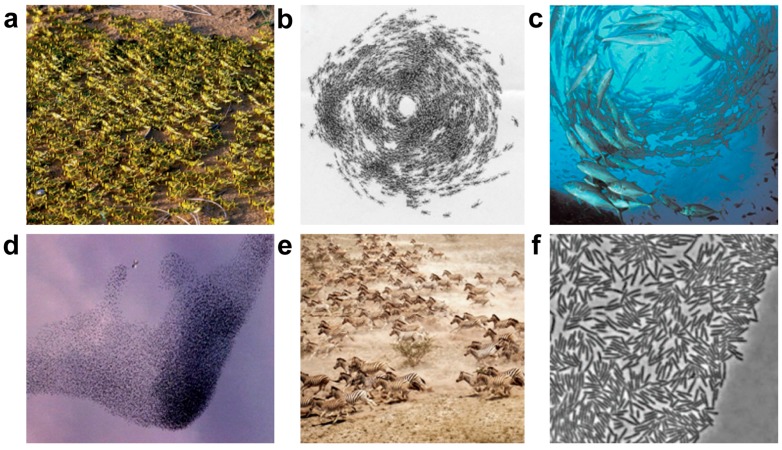
Collective behavior in nature. (**a**) Locusts marching. (**b**) Colony of army ants. (**c**) Fish vortices. (**d**) Thousands of birds producing a fascinating display. (**e**) A herd of zebra. (**a**–**d** reproduced from [2]) (**f**) Swarming of Escherichia coli (reproduced from [37]).

The macroscopic collective behavior of natural animals, such as a herd of land animals, locusts marching, a flock of birds, a colony of army ants, or a school of fish, is a familiar and beautiful part of the living system (Figure 1a–e) [2,35]. The formation of such collective behaviors may be a consequence of an external stimulus or the local interactions between nearby individuals. Taking a school of fish as an example, each individual complies with its behavior on its position and nearest velocity of neighbors. Hundreds of small silver fish glide in unison, much like a single organism than a collection of individuals. Even not a single fish is lost from the group or suddenly change the direction. Such interactions results in a global collective behavior that may emerge, originating and maintaining the cohesion of the aggregate [35,36].

In the microscopic world, collective behavior of cells and bacteria (Figure 1f) is a common phenomenon that occurs on everybody. For bacteria, many genera of motile bacteria use a mechanism, referred to as ‘swarming or collective’, to access new sources of nutrients at surface, and to increase the size of the colony [37]. Taking cells into consideration, the migration of cells may respond to the surrounding environment and interacts between other cells through physical contact and soluble substrates, and change their behaviors accordingly.

## 3. Controlled Swarming and Assembly of Artificial Micromotors

Much like biological systems, micro/nanomachines also respond to external gradients and exhibit schooling and exclusion behaviors. These gradients can be achieved by several approaches, including chemical, electrical, light, ultrasound, and magnetic. Dependent on interparticle attraction and exclusion, and reactions between the gradient and nanomotors, such as catalytic reaction, dielectrophoresis, electromegnetic reaction and etc., controlled swarming and assembly of artificial micromotors can be realized. Such assembly of micro/nanoparticles is of important to fabricate materials with multifunctional properties (e.g., mechanical, conductive, and optical) and to accomplish complex tasks [38,39,40]. In the following sections, based on the different external gradients, we will discuss the main approaches that can induce the collective behavior of micro/nanomachines and their related applications.

### 3.1. Chemical Induced Collective Behavior

**Figure 2:**
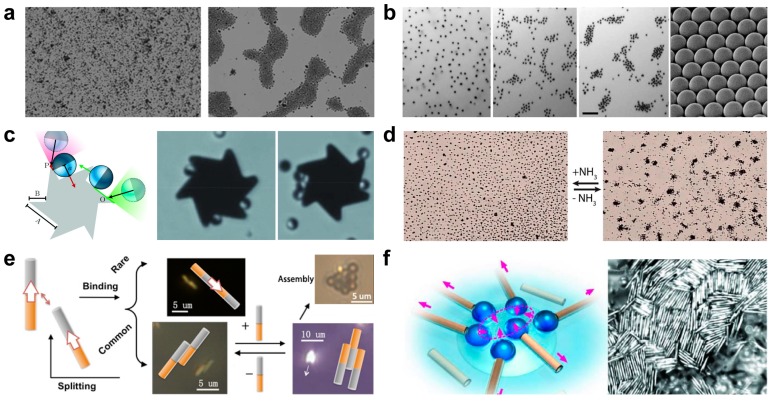
Chemical induced swarming and assembly of micro/nanoparticles. (**a**) Reversible swarm formation of unmodified Au microparticles induced by repetitive hydrazine additions (reproduced from [41]). (**b**) The swarming behavior of the Ir-based micromotors (reproduced from [42]). (**c**) Self-Assembly of micromachining systems powered by Janus micromotors (reproduced from [43]). (**d**) Transition between exclusion and schooling behaviors based on self-diffusiophoresis (reproduced from [44]). (**e**) Catalytically powered dynamic assembly of rod-shaped nanomotors and passive tracer particles (reproduced from [47]). (**f**) Self-assembled tubular microengines (reproduced from [48]).

Micro/nanomotors tend to move preferentially in the direction of externally applied chemical gradient, which is defined as chemotaxis. The chemical induced assembly almost exists in all types of catalytic micro/nanomachines, ranging from a simple spatial geometry, such as Janus spheres, Au-Pt nanowires, or microtubes to complex large units [3,32]. Chemotaxis has been observed in self-propelled unmodified Au microparticles (0.8–1.5 μm) by Joseph Wang’s group [41]. As demonstrated in Figure 2a, the reversible swarming of Au microparticles can be achieved by repetitive addition of hydrazine. The possible mechanism of such chemically triggered swarming of Au microparticles was the diffusion of ionic products from the catalytic Au surface. The reaction between hydrazine and H_2_O_2_ catalyzed by the gold surface generated local amount of anions and cations, including H^+^, N_2_H_5_^+^, and OH^−^, and generated chemical gradients on the nearby Au microparticles, which pulled the particles towards the center of the swarms where the largest electrolyte gradient existed. The same group also reported chemical induced assembly of Ir/SiO_2_ Janus nanomotor, as demonstrated in Figure 2b [42]. When compared with previously mentioned assembly of Au microparticle (Figure 2a), the Ir/SiO_2_ Janus nanomotor (1.2 μm diameter) displayed a collective swarming behavior in response to their environment autonomously with the sole presence of extreme low levels of hydrazine fuel (only 0.001%). The collective of Janus micromotors (1.2 μm diameter) to propel a six asymmetric teeth (with an external radius of 8 μm) was reported by Leonardo’s group, as demonstrated in Figure 2c [43]. The active Janus microparticles were made of platinum half-coated on silica particles (Diameter: 5 μm), and could be propelled in a hydrogen peroxide solution. Self-generated solute gradient was based on dynamical behavior of Janus particles colliding with solid obstacles, the approaching walls particles that were assembled on the surface and oriented their symmetry axis parallel to the wall surface, and finally propelled the six asymmetric teeth.

Self-assembled chemical structures produce molecular aggregates with complex functionalities, which are often inspired by biological systems. While self-assembly has been the focus of intense investigation, the disassembly of these molecular aggregates is much less explored, even though it can lead to temporal control over the aggregate functionality. Sen’s group reported a reversible Ag_3_PO_4_ microparticle system showing collective behaviors and disassembly in (or without) NH_3_ medium in Figure 2d [44]. The schooling behavior of Ag_3_PO_4_ microparticles was based on self-diffusiophoresis. When adding NH_3_, the production of large numbers of OH^−^ resulted in faster diffusion away from the Ag_3_PO_4_ microparticles than the other ions, and led to exclusionary behavior. When removing NH_3_, the reaction was reversed, and schooling behavior was observed. The same group also reported an enzyme pump that nanomotors migrated towards regions of higher substrate concentration. The surface-immobilized enzymes, independent of adenosine triphosphate, were functioned as self-powered micropumps in the presence of their respective substrates. The flow was driven by a gradient in fluid density that was generated by the enzymatic reaction. Such behaviors could autonomously deliver proteins and assemble particles in response to specific chemical stimuli [45,46].

Au-Pt nanowires that were powered by hydrogen peroxide, also show collective reaction when placed close to each other. Wang et al. studied the mechanism of the interaction of two nanowires as shown in Figure 2e [47]. The numerical simulations of interactions between two Au-Pt nanowires in hydrogen peroxide solutions revealed that the attractive and repulsive interactions were generated in the process of catalytic decomposition of hydrogen peroxide. Electrokinetic effects drove the two assembled doublets moving together in the same direction. Such behavior cannot be observed when nanowires composed of only gold or platinum. Schmidt’s group studied the collective behavior of catalytic tubular microengines, as sketched in Figure 2f [48]. The catalytic microtubes can attract each other by bubbles at air-liquid interface and can self-assemble into compact patterns due to the meniscus-climbing effect, as shown in Figure 2f right. Such a balanced system offers an intriguing way to study dynamic self-assembly of bubble-propelled microengines.

Chemical gradient provides an approach to study the autonomous collective behavior, and can trigger swarming behavior of Janus spheres, Au-Pt nanowires, or microtubes into complex large units to achieve multifunctional properties. However, it should be noted that most of the aforementioned chemical triggered swarming behaviors are based on the interaction between the micro/nanomachines and the medium, which makes it not easily reversible and controllable. Besides, the swarming time is highly dependent on the reaction speed that makes it much longer than other approaches.

### 3.2. External Field Induced Collective Behavior

Besides chemotaxis, several external physical stimuli (for example, optical, electric, ultrasound, and magnetic fields) were used to induce the swarming of microparticles. When compared with chemotaxis, external physical stimuli induced assembly has shown promising performance with major advantages of on-demand motion control. The following sections will discuss recent progress that has been made in on-demand triggering the swarming or collective behavior of micromachines in the past decade.

#### 3.2.1. Electric Induced Collective or Assembly Behavior

**Figure 3:**
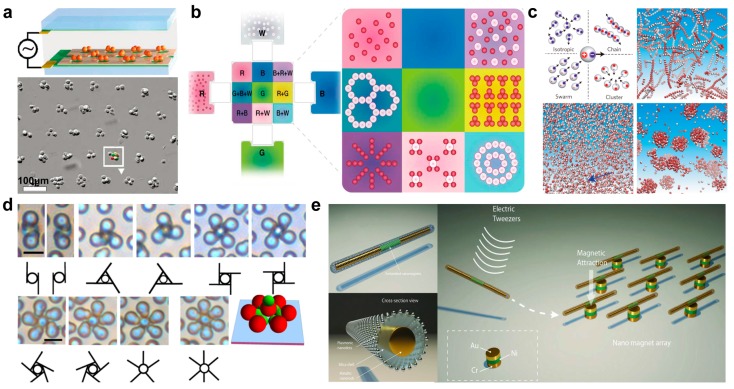
Electric field induced swarming and assembly of micro/nanoparticles. (**a**) Patterned electrode for multiply cell patterns (reproduced from [49]). (**b**) Programmable electromicrofluidic platform for microgel formation and architecture assembly (reproduced from [50]). (**c**) The strategy is to program dual electric charges shifted from the sphere center onto opposing hemispheres (reproduced from [51]). (**d**) Electric-field-induced assembly and propulsion of chiral colloidal clusters (reproduced from [52]). (**e**) Electric tweezers (reproduced from [53]).

Electric induced dielectrophoresis, is a phenomenon exerted on dielectric micro/nanoparticles suspended in water solution when the micro/nanoparticles exist in a nonuniform electric field. Such phenomenon can be used to trigger the assembly of cells. As demonstrated in Figure 3a [49], applying alternating current on a patterned SU-8 on indium tin oxide (ITO)-coated glass slides resulted in a spatially patterned electric field, and led to the propelling the cells toward circular gaps, where the high intensity electric field was located and forming the two-dimensional (2D) patterns. Such behavior is greatly helpful in cell functions research. By combining electric manipulation with microfluidic, Chiang et al. demonstrated electro-microfluidic platform for microgel formation and architecture assembly (Figure 3b) [50]. The conceptual electro-microfluidic platform employed four cross-linkable elementary materials to assemble an architecture consisting of 3 × 3 microgels. The formation of patterned microgels was based on the manipulations of suspended particles, liquid droplets, and crosslinked microgels by electrowetting and dielectrophoresis between parallel plates with appropriate electrodes and dielectric and hydrophobic layers.

Reconfiguring assembly of active Janus particles by electrostatic imbalance was achieved by Yan et al. [51]. A three-dimensional (3D) simulation shown in Figure 3c, the different electric charges on two end of Janus particles could result in dramatic assembly behavior. Janus microswimmers with equal-and-opposite charges on each hemisphere can polymerize into connected chains; if the charge on one hemisphere greatly exceeded that on the other, a torque arose when Janus microswimmer came into proximity. This torque diverted head-repulsive swimmers to avoid head-to-head collisions, and resulted in two-Janus particle alignment, and led to the collective or swarming of particles on a large scale; when tail-repulsive particles in proximity were preferentially positioned their tails apart, they were encouraged to face each other and to form jammed clusters with high local density. The dynamic reversible assembly of Janus microparticles was also reported by Wu’s group, as shown in Figure 3d [52]. The geometry of the assembly depended on both the geometry and orientation of Janus particles. The assembly can be trimer, tetramer, and pentamer, with an increasing number of petals. The author also checked the tetramers formed from dimers with different aspect ratios. When electric field turned off, the Janus particles could disperse. This strategy of electric-controlled collective assembly illustrated more general principles, holding great potential in fabricating 2D or 3D materials.

Combine DC with AC electric fields to manipulate nanowires suspended in a liquid was reported by Fan’ group, called electric tweezers [53]. The electric tweezer always consisted of an Au/Ni/Au nanowire as rotor and an Au/Ni/Cr nanodisk as bearing. The Au/Ni/Au nanowire can attach on the patterned Au/Ni/Cr bearing by magnetic interaction, as shown in Figure 3e [54]. The patterned nanowires motor was controllable and can be rotated under a DC and AC electric field at an ultrafast speed of 18,000 r.p.m [55]. Due to the easy-controllability of such manipulation of a nanowire, by modifying the nanowires with a cytokine, such as tumour-necrosis factor-alpha, same group demonstrated a single cell drug delivery [56].

When compared with chemical induced collective behavior, the electric induced swarming behavior shows advantage of on-command controllability. However, it should be noted that the aforementioned electrically triggered patterning micro/nanoparticles always require complex patterned electrodes to generate 2D plane electric field. The swarming effects are highly dependent on the properties of suspended micro/nanoparticles (dielectric or conductive) and the high voltage of electric field may cause damages to cells, which greatly limit their application towards in vivo studies.

#### 3.2.2. Collective or Assembly Behavior of Magnetic Micro/Nanoswimmer

**Figure 4:**
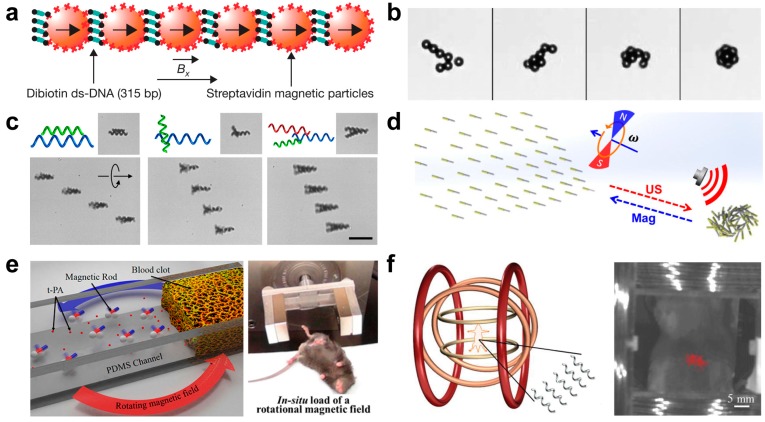
Magnetic field induced swarming and assembly of micro/nanoparticles. (**a**) Magnetic field B_x_ guided the particles modified with streptavidin to interact with biotin-modified double-stranded DNA and to form filaments via the specific biotin-streptavidin interaction (reproduced from [64]). (**b**) Rotating magnetic field B_x_ + B_y_ for colloids assembly (reproduced from [65]). (**c**) Magnetically assisted assembly and disassembly of microhelices (reproduced from [66]). (**d**) Complex spatial-temporal collective behaviors of magneto-acoustic hybrid nanomotors (reproduced from [67]). (**e**) Rotating magnetic nanomotors enhanced the mass transport of t-PA molecules at the blood clot interface for local ischemic stroke therapy (reproduced from [68]). (**f**) Controlled swimming of a swarm of artificial bacterial flagella in the intra peritoneal cavity of a Balb-C mouse (reproduced from [69]).

Magnetism is one of the most efficient and effective ways to remotely control the motion of synthetic micro/nanomachines. Several individual groups have introduced ferromagnetic segments (Ni, Co, or Fe) to navigate micro/nanomachines to achieve certain tasks [57,58,59,60]. Recently, the magnetic induced assembly of micro/nanomachines attracted much attention for multiply functions [61,62,63]. As schematized in Figure 4a, movement in pattern (filaments) can be achieved under an applied magnetic field B_x_. Such filament was constructed by double-stranded DNA with biotin at each end that can bind the particles together via the specific biotin-streptavidin interaction. The actuation induced a beating pattern that propelled the structure, and the external fields can be adjusted to control the velocity and the direction of motion [64]. Applying magnetic field to assemble colloidal microwheels was achieved by Tasci et al., as shown in Figure 4b [65]. The microwheels can be assembled from individual colloidal under rotating magnetic fields in both B_x_ and B_y_ direction of magnetic field at the surface plane. Reversible assembly of wheel-shaped devices can also be achieved, and microwheels can be directed rapidly and precisely along user-defined paths by varying spin frequency and angle relative to the surface.

The schooling behavior of magnetic helical micromotors was investigated by Tottori et al. as shown in Figure 4c [66]. The applied rotating magnetic forces can induce the assembly and disassembly of two swimmers to from a chain configuration. Multiple microswimmers can configure various geometrical configurations including straight, bent chains, and crosses. The speed of the resulting assemblies was highly dependent on their geometrical configurations. Such assembly behavior of individual is greatly helpful for particular designs and configurations. Reversible assembly of magneto-acoustic hybrid nanomotors was reported by Li et al., as demonstrated in Figure 4d [67]. Under the manipulation of both ultrasound and magnetic, the hybrid nanomotors can be regulated into three dramatic forms as follows. Only magnetic field on, directional moving individual nanomotors was observed. Only ultrasound field on, rapid migration of the nanomotors toward the original node position was observed. Both ultrasound and magnetic on, reformation of a dynamic swarm vortex was observed.

The ultimate goal of micro/nanomotors is towards in vivo medical application. Zhao’s group reported an approach that accelerated tissue plasminogen activator-mediated thrombolysis by magnetically powered colony nanomotors, as demonstrated in Figure 4e [68]. The Ni-rod nanomotors fabricated by glancing angle deposition approach, and then coated a layer of biocompatible and biodegradable poly (lactic-co-glycolic) aggregates composed of t-PA-coated nanoparticles. Rotating magnetic nanomotors could enhance the mass transport of t-PA molecules at the blood clot interface for local ischemic stroke therapy. Such micro-aggregates can hold the nanoparticles together for enhancing the treatment of thrombolysis. Controlled swimming of a swarm of artificial bacterial flagella (16 μm in length) in Balb-C mouse under 9 mT and 90 Hz was reported by Nelson’s group [69]. Due to the florescence of near-infrared probes, whole-body in vivo optical imaging of a swarm of functionalized artificial bacterial flagella was achieved by the magnetically controlled navigation, as shown in Figure 4f. The above mentioned in vivo study of micro/nanomachines hold great potential in future transport in therapeutics and microsurgery.

Magnetism is the most widely used method to remotely trigger and control the swarming behavior of micro/nanoparticles with great biocompability. Although there is still plenty of room for improvement, scientists have already begun to use this approach towards preliminary biological applications. However, such an approach always requires the ferromagnetism of the micro/nanoparticle, which may limit some application prospects.

#### 3.2.3. Ultrasound Induced Collective or Assembly Behavior

**Figure 5:**
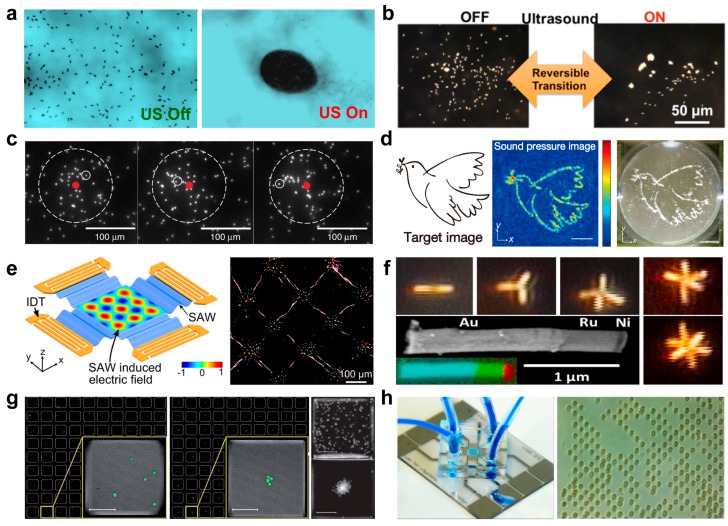
Ultrasound induced swarming and assembly of micro/nanoparticles. (**a**) Ultrasound swarming of Au-Pt catalytic nanowires (reproduced from [71]). (**b**) Reversible swarming behavior of Au-Ru microrods (reproduced from [72]). (**c**) Acoustic trapping of Janus Pt/PS particles (reproduced from [73]). (**d**) Holograms for acoustics (reproduced from [11]). (**e**) Tunable nanowire patterning using standing surface acoustic waves (reproduced from [74]). (**f**) Self-assembly of nanorod motors into geometrically regular multimers and their propulsion by ultrasound (reproduced from [75]). (**g**) Ultrasound-controlled cell aggregation in a multi-well chip (reproduced from [76]). (**h**) Surface acoustic wave for assembly of single cell (reproduced from [77]).

Ultrasound is another efficient tool to trigger the assembly or swarming of micro/nanoparticles [70]. Ultrasound manipulation of micro/nanomachines is based on pressure nodes or antinodes, which are responsible for particles migration or collection in certain areas. The using ultrasound field to regulate the swarming behavior of Au-Pt nanomotors hold considerable promise for fabricating man-made nanomachines that mimics the swarming behavior of natural animals. Ultrasound standing waves triggered swarming behavior of Au-Pt nanomotors (200 nm in diameter, 2 μm in length) was reported by Xu et al., as demonstrated in Figure 5a [71]. Without ultrasound field, Au-Pt nanomotors catalyzed decomposition of hydrogen peroxide and displayed autonomous random motion, as shown in Figure 5a left. With ultrasound field, Au-Pt nanomotors fast migrated towards ultrasound pressure nodes and then rapidly formed a swarm, as shown in the right of Figure 5a. The same behavior also reported by Wang et al., as shown in Figure 5b [72]. Transition between aggregated and dispersed states of a group of Au-Ru nanowires (~3 μm long and ~300 nm in diameter) can be found at the bottom of acoustic cell. Ultrasound-triggered reversible swarming of Janus microparticles (2 and 3 μm in diameter) also reported, as demonstrated in Figure 5c [73]. When in a weak acoustic trap, the Janus microswimmers were allowed to explore and reorient freely before reaching the ‘ends’ of the well. When in a strong acoustic trap, the Janus microswimmers can be fully trapped into a swarm, and then spread diffusively after turning off the ultrasound field. Such ultrasound triggered reversible swarming of micromachines may give insight into the origin of polar order, how and why living organisms align.

Ultrasound patterned regular geometric graphs have been reported by several groups. Acoustic hologram behavior was investigated, as shown in Figure 5d [11]. The acoustic holograms introduced a monolithic acoustic hologram by using a simple planar transducer driven by a single function generator and amplifier. By modifying the output of a single ultrasonic transducer, a designed 2D phase profile that is generated by the assembled particles can be achieved. Such acoustic induced holograms hold great potential in rapid fabrication of complex sound fields appropriate for super-resolution imaging, selective heating, and personalized medicine. The use of standing surface acoustic waves to trigger nanowires patterning was demonstrated in Figure 5e [74]. Two pairs of parallel interdigital transducers were deposited on a piezoelectric substrate to generate 2D standing surface acoustic waves. With a consistent spacing of half of the wavelength, the polystyrene microspheres were driven to the displacement antinodes, where the nanowires experienced counter-balancing forces from both positive and negative electric charges. Such a hypothesis was also confirmed by adding both silver nanowires and polystyrene microspheres in the 2D standing surface acoustic waves to observe their relative locations. Ultrasound-triggered the self-assembly of nanorod motors into geometrically regular multimers was reported by Mallouk’s group [75]. As demonstrated in Figure 5f, Au-Ru-Ni nanowires (300 nm in diameter, 2.0 μm in length) can assemble into geometrically regular dimers, trimers, and higher multimers under a ~4 MHz of ultrasound at the midpoint of a cylindrical ultrasound cell. Such assembly can be propelled autonomously in fluids by excitation with ~4 MHz of ultrasound and exhibit several distinct modes of motion.

Ultrasound tweezers were also used for focusing or patterning of microparticles and cells, as shown in Figure 5g,h [76,77]. Cell lines, 2D cell patterns, and single cell patterns can be achieved by changing the pair numbers of parallel interdigital transducers and using the appropriate frequency. In details, when one pair of parallel interdigital transducers were applied, multiply cell lines can be obtained [78]. As the number of parallel interdigital transducers increased to two pairs, two dimensional ultrasound potential can be achieved, resulting in the generation of a 2D cell patterns [79]. In addition, by adjusting the ratio of λ/D (wavelength/cell diameter), 2D single cell patterns can be obtained [77].

Ultrasound is the fastest and biocompatible approach to trigger the swarming behavior of micro/nanomachines. Theoretically, such an approach could trigger all kinds of micro/nanoparticles without dielectric or magnetic properties. Until now, such approach has achieved some proof-of-concept biological applications, more efforts must be devoted to fabricating more precisely acoustic device to achieve complex in vivo targeted drug delivery studies.

#### 3.2.4. Light Induced Collective or Assembly Behavior

**Figure 6:**
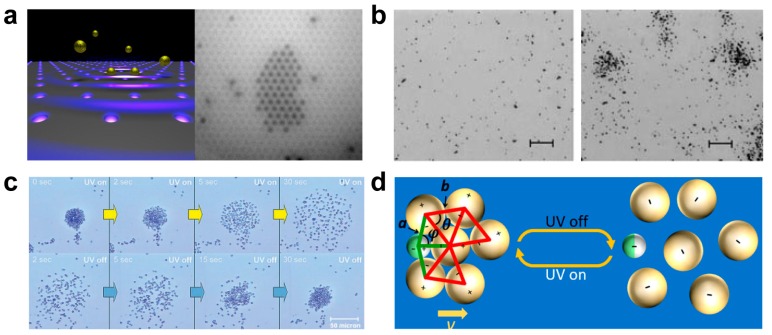
Light induced swarming and assembly of micro/nanoparticles. (**a**) Optical analogue of epitaxial growth to assemble gold nanoparticles (reproduced from [86]). (**b**) Schooling behavior of light-powered AgCl micromotors (reproduced from [87]). (**c**) Titanium-dioxide-based reversible micropump systems (reproduced from [88]). (**d**) TiO_2_/Pt Janus micromotors with light irradiation for reversible and in-place assembly dissociation of surrounding SiO_2_ particles (reproduced from [89]).

Light is also a powerful tool to study the swarming or collective behavior of the micro/nanomachines. Beside aforementioned electric tweezers and ultrasound tweezers, the use of a strongly focused light beam to precisely trap and manipulate micro/nanoscale objects is called optical tweezers. Micro/nanoscale objects can be polarized by intensity gradients that are generated by a converging beam, and then move toward the highest gradient region of the electric field. Optical tweezers can trap micro/nanoparticles vary from two dimensions to three dimensions, from a single object to multiple objects [80,81,82,83,84]. Light-assisted self-assembly of gold nanoparticle chains has been reported [85]. Optical tweezers patterned highly stable 2D array of Au nanoparticles (diameter: 200 nm) was shown in Figure 6a [86]. The Au nanoparticles can be precisely patterned with a uniform space of 1.1 μm. Such optical tweezers induced 2D pattern is a new kind of template-free and reconfigurable method to reconfigure colloidal crystals.

Ibele et al. demonstrated that silver chloride (AgCl) microparticles interacted with each other at high concentrations and formed schools in presence of UV light, as shown in Figure 6b [87]. AgCl microparticles (1 μm in diameter) randomly scattered in deionized water before UV illumination (Figure 6b left). After 90 s of UV irradiation, asymmetric photodecomposition of AgCl microparticles resulted in electrolyte gradient, and led to the collective behavior of AgCl microparticles (Figure 6b right). Similar behavior can also be observed with titanium dioxide (TiO_2_) microparticles (size range 0.2–2.5 µm), as reported by the same group, as shown in Figure 6c [88]. Under UV illumination, the TiO_2_ microboats decomposed water and generate electrolyte gradient to trigger the collective behavior of surrounding silica microspheres (2.34 µm), which was unlike AgCl microparticles that photo-decomposed itself to produce the movement.

The light-triggered reversible swarming behavior of Janus microparticles was reported by Palacci et al. [90]. Under the illumination of blue light, such Janus microparticles catalyzed the exothermic chemical decomposition of H_2_O_2_, creating chemical gradients, and resulted in assembly into a homogeneous compact pattern. In contrast, the microparticles presented an incompact pattern when the light was off. Guan’s group also reported an approach that in-place assembly dissociation in a high accuracy by manipulating TiO_2_/Pt Janus micromotors with light irradiation (Figure 6d) [89]. Under UV irradiation, TiO_2_/Pt Janus micromotors generated electron-hole pairs and involved in the oxidation of H_2_O_2_, producing a chemical gradient. Such a gradient can result in the assembly of surrounding SiO_2_ colloidal particles. Visible light-triggered micropump for microparticle assembly was reported by Esplandiu et al. [91]. The micropump was simply fabricated by using standard electron beam lithography and electron beam evaporation, which consisted of 30–50 μm diameter Pt disks on doped silicon wafers. The application of light can lead to photoactivated chemical reactions at the silicon and the metal surfaces, which generated a chemical gradient, and resulted in such collective behavior.

Light has been the most convenient approach to induce the collective or assembly behavior due to its no requirement of complex processes for device fabrication. However, there are also some limitations. For the optical tweezers, the dielectricity of nanoparticles could result in serious local heating problems existing at the optical traps due to the high intensity of the focused laser beam. The photothermal and photocatalytic collective mechanisms always require some special materials (photothermal or photocatalytic properties) that may limit their practical application. Besides, the requirement of a certain degree of transparency in such systems also make it challenging towards clinical studies.

## 4. The Comparison of Different Approaches

Chemical induced collective behavior is based on the reaction on surface, so it is highly related to the surface properties, regardless of the dimensions and morphology. Chemical-induced assembly is quite simple, however, it does not have the capability of disassembly. For now, it is far away for chemical induced swarming behavior to be a tunable control approach. The response time is a little long, which requires a few minutes. The reaction are always carried out in water solution medium. The adding of extra fuel (H_2_O_2_, N_2_H_4_, etc.) could be a bit of toxicity towards in vivo studies.

Electric induced collective behavior is based on the interaction between the electric and the microparticle. No special nature and morphology of the particles is required. Electric always require designing the device to be suitable for known particle size. The electric induced assembly behavior could be reversible if the microparticle is alive. The controllability of electric swarming behavior is quite easy, microparticles can be controlled as regular patterns or directional arrangement. The response time is short, which requires only a few seconds. The reaction is often in solution medium, and it is not easy to operate towards in vivo study, because the high voltage may damage the tissue.

Magnetic induced collective behavior is based on the interaction between magnet and ferromagnetic micro/nanoparticles. This mechanism introduce ferromagnetic materials, such as Iron, Cobalt, and Nickel into the micromachines, no special morphology or dimension is required. Magnetic induced collective behavior is a tunable control approach and has the capability of disassembly by controlling the magnetic field. The response time is just a few seconds. The operation can be superfast and can be applied in almost all solution medium without any toxicity.

Ultrasound induced collective behavior is based on the interaction between suspended foreign particles and the applied ultrasound filed. No special nature or morphology or dimension is required. The ultrasound induced collective behavior can be a tunable control and fully reversible by changing the applied acoustic frequency or using active micro/nanoparticles. The response time is just several seconds and can be applied in almost all solution the mediums, without any toxicity. However this approach requires relatively complex fabrication processes to produce specific acoustic waves.

The optical tweezers can only manipulate the micro/nanoscale objects with dimensions smaller than the optical wavelength (few nanometers to hundred nanometers), and most of the morphology of the objects is spherical. It is a fully controllable approach with a short response time, and it can be applied in certain transparent solution medium without any toxicity. For the light-induced chemical reaction collective behavior, the detail is much like the chemical induced collective behavior. The only difference is the requirement of photothermal or photocatalytic material in the micro/nanomachines.

## 5. Conclusions and Future Directions

Self-organization of natural animals can build complex forms, from multicellular organisms to complex animal structures, such as flocks of birds and schooling of fishes by the interaction of individuals. Although tremendous efforts have been devoted to creating this ability in controlling the swarming or collective behavior of micro/nanomachines by chemical, electrical, magnetic, ultrasound, and light, much challenges must be faced in the design of both programmable and intelligent systems that can operate at microscale or nanoscale. The future direction of the swarming of micro/nanomachines is to create a system that demonstrates programmable self-assembly of thousand-micromachines into complex three-dimensional shapes for certain tasks. With the development of controllable swarming or collective behavior of micro/nanomachines, self-organized systems could be swarm intelligence. Swarm intelligence typically consists of a population of simple units interacting locally with one another and with their environment. The individual units will follow very simple rules, simple control of such a swarm can lead to the emergence of “intelligent” global behavior. This motivates new investigations into advanced collective micro/nanomachines to recover large-scale external damages and to accomplish much complex in vivo or vitro biomedical applications.
